# Supplementary material for: Splice-Junction-Based Mapping of Alternative Isoforms in the Human Proteome
Source: Cell Rep. Author manuscript; Available in PMC 2020 Jan 15. (PMC6961840; doi:10.1016/j.celrep.2019.11.026)
Supplement: 3 [file NIHMS1546469-supplement-3.zip › DF2/PXD0010154/Testis-219-Q15811-VASPAAKPVVSGEEIAQVIAS.pdf]

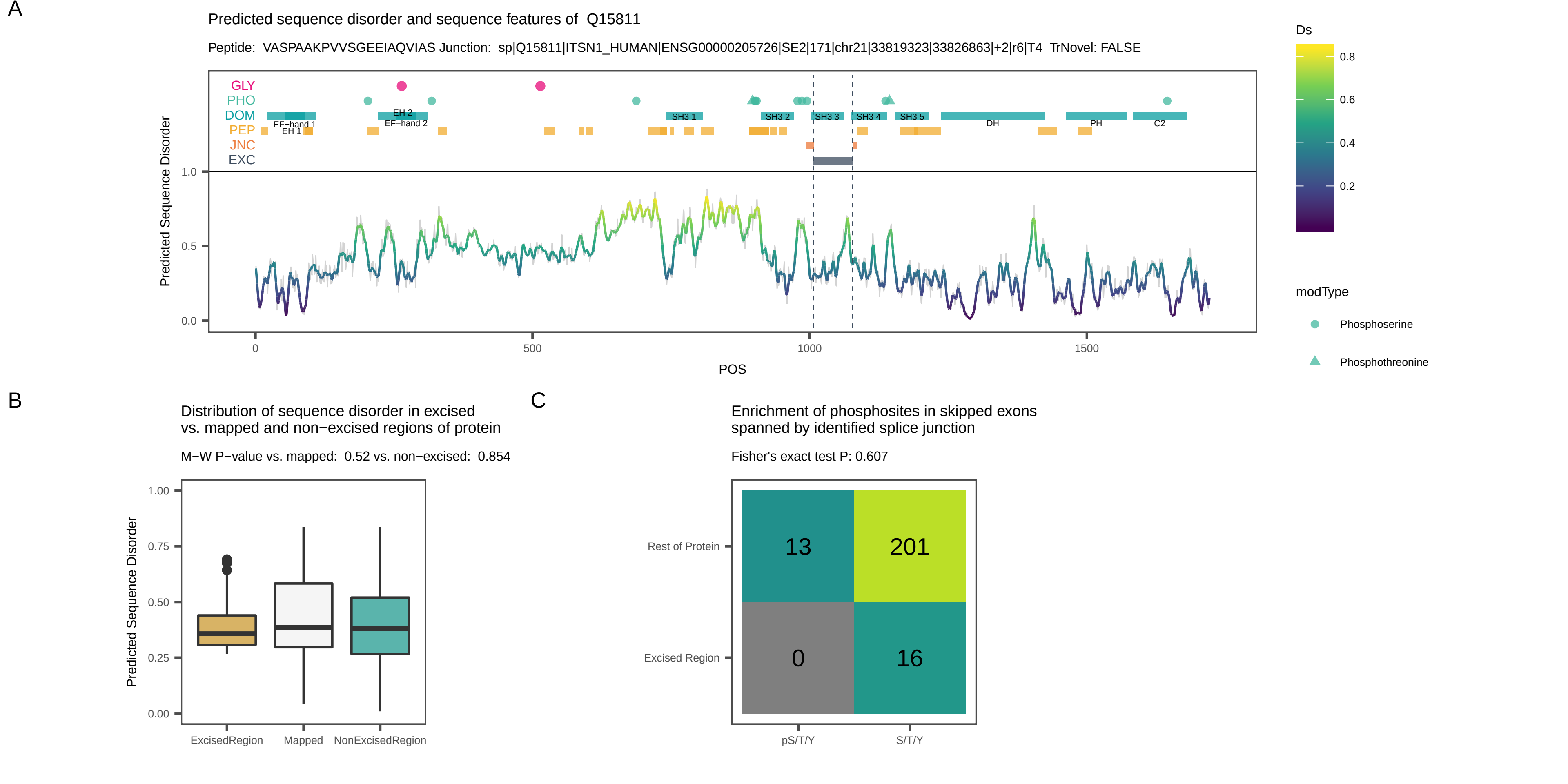
A
Predicted sequence disorder and sequence features of Q15811
Peptide: VASPAAKPVVSGEEIAQVIAS Junction: sp|Q15811|ITSN1_HUMAN|ENSG00000205726|SE2|171|chr21|33819323|33826863|+2|r6|T4 TrNovel: FALSE
GLY
PHO
DOM
PEP
JNC
EXC
EF−hand 1
EH 1
EH 2
EF−hand 2
SH3 1
SH3 2
SH3 3
SH3 4
SH3 5
DH
PH
C2
Predicted Sequence Disorder
1.0
0.5
0.0
0
500
1000
1500
POS
Ds
0.8
0.6
0.4
0.2
modType
Phosphoserine
Phosphothreonine
B
Distribution of sequence disorder in excised
vs. mapped and non−excised regions of protein
M−W P−value vs. mapped: 0.52 vs. non−excised: 0.854
Predicted Sequence Disorder
1.00
0.75
0.50
0.25
0.00
ExcisedRegion
Mapped
NonExcisedRegion
C
Enrichment of phosphosites in skipped exons
spanned by identified splice junction
Fisher's exact test P: 0.607
Rest of Protein
Excised Region
13
201
0
16
pS/T/Y
S/T/Y
